# Effects of robotic-assisted laparoscopic prostatectomy on surgical pathology specimens

**DOI:** 10.1186/1746-1596-7-24

**Published:** 2012-03-13

**Authors:** Heng Hong, Lin Mel, Jonathan Taylor, Qiang Wu, Hugh Reeves

**Affiliations:** 1 Brody School of Medicine at East Carolina University, Greenville, NC, USA; 2 Eastern Urological Associates, P.A., Greenville, NC, USA; 3 College of Allied Health Sciences at East Carolina University, Greenville, NC, USA

**Keywords:** Robotic-assisted laparoscopic prostatectomy, Radical retropubic prostatectomy, Prostate cancer, Positive surgical margin, Capsular incision

## Abstract

**Background:**

Robotic-assisted laparoscopic prostatectomy (RALP) has greatly changed clinical management of prostate cancer. It is important for pathologists and urologists to compare RALP with conventional open radical retropubic prostatectomy (RRP), and evaluate their effects on surgical pathology specimens.

**Methods:**

We retrospectively reviewed and statistically analyzed 262 consecutive RALP (n = 182) and RRP (n = 80) procedures performed in our institution from 2007 to 2010. From these, 49 RALP and 33 RRP cases were randomly selected for additional microscopic examination to analyze the degree of capsular incision and the amount of residual prostate surface adipose tissue.

**Results:**

Positive surgical margins were present in 28.6% RALP and 57.5% RRP cases, a statistically significant difference. In patients with stage T2c tumors, which represent 61.2% RALP and 63.8% RRP patients, the positive surgical margin rate was 24.1% in the RALP group and 58.8% in the RRP group (statistically significant difference). For other pathologic stages, the differences in positive margins between RALP and RRP groups were not statistically significant. The incidence of positive surgical margins after RALP was related to higher tumor stage, higher Gleason score, higher tumor volume and lower prostate weight, but was not related to the surgeons performing the procedure. When compared with RRP, RALP also caused less severe prostatic capsular incision and maintained larger amounts of residual surface adipose tissue in prostatectomy specimens.

**Conclusions:**

In this study RALP showed a statistically significant lower positive surgical margin rate than RRP. Analysis of capsular incision and amount of prostatic surface residual adipose tissue suggested that RALP caused less prostatic capsular damage than RRP.

**Virtual slides:**

The virtual slide(s) for this article can be found here: http://www.diagnosticpathology.diagnomx.eu/vs/1278078279667611

## Background

Prostate cancer is the most common cancer and the second leading cause of cancer-related death in men in the United States [1]. Open radical retropubic prostatectomy (RRP) has long been the standard surgical procedure for the treatment of localized prostate cancer. In 1997, Schuessler et al. reported the first successful laparoscopic radical prostatectomy in an effort to reduce the morbidity of open radical prostatectomy [2], but because of its steep learning curve, laparoscopic radical prostatectomy was not widely adopted by most urologic surgeons in the United States. In 2000, Binder and Kramer performed the first robotic-assisted laparoscopic prostatectomy (RALP), which allowed three-dimensional viewing, improved ergonomic efficiency, eliminated hand tremor, and refined dexterity [3]. With the introduction of advanced robotic devices such as the Da Vinci Surgical System (Intuitive Surgical, Inc., Sunnyvale, CA), RALP has been so widely accepted in the treatment of prostate cancer that it is currently utilized in an estimated 69-85% of prostatectomies in the United States [4]. Compiled data has shown that in high-volume centers RALP is a safe option for treatment of patients with localized prostate cancer, with similar overall complication rates as conventional RRP. In some studies, RALP showed lower operative blood loss, decreased need for transfusion, and higher continence and potency rates when compared with RRP [5]. However, comparison of these procedures is still limited by the lack of randomized trials and long-term follow-up studies.

Because the long-term data comparing biochemical recurrence and disease-free survival between RALP and RRP are still not available due to the relatively short history of RALP, the incidence of positive surgical margins after RALP has become important in the evaluation of oncological outcomes of this procedure. In previous studies based on the analysis of RRP, positive surgical margins were found to be associated with an increased risk of biochemical and local disease recurrence, as well as the need for secondary treatment after radical prostatectomy [6]. The incidence of positive surgical margins after prostatectomy is related to many factors such as the preoperative serum prostate-specific antigen (PSA) level, Gleason score, and tumor volume [7]. Differing surgical procedures may also contribute to the varying incidence of positive surgical margins; therefore, in this study we analyzed prostatectomies performed in our institute from 2007 to 2010, in an effort to compare RALP with conventional RRP. We compared the positive surgical margin rates between these two procedures and analyzed the factors associated with the incidence of positive surgical margin in RALP. Moreover, in order to evaluate the prostate capsular damage caused by both surgical procedures, we analyzed capsular incision and residual adipose tissue on the prostatic surface after RALP and RRP.

## Materials and methods

### Patient population

This study analyzed 262 consecutive prostatectomies, including 182 cases of RALP and 80 cases of RRP, performed in Pitt County Memorial Hospital in Greenville, North Carolina, during the period of August 2007 to March 2010. All the prostatectomy procedures were performed by surgeons from Eastern Urological Associates, P.A., Greenville, North Carolina. The da Vinci Surgical System (Intuitive Surgical, Inc., Sunnyvale, CA) was used in RALP procedures. This study conformed to the Helsinki declaration and was approved by the University and Medical Center Institutional Review Board of East Carolina University.

### Pathological processing of the prostatectomy specimens

All the prostatectomy specimens were processed in the Department of Pathology and Laboratory Medicine of the Brody School of Medicine at East Carolina University. Surgical pathology reports were signed out by board-certified pathologists, based on the findings from gross and microscopic examinations. This study collected and retrospectively examined the surgical pathology reports of 262 cases of RALP and RRP from the file in the pathology department. The data collected from surgical pathology reports were statistically analyzed.

### Evaluation of prostatic capsular incision and surface adipose tissue

Eighty-two cases of prostatectomies, including 49 RALP and 33 RRP, were randomly selected for additional microscopic examination to evaluate prostatic capsular incision and the presence of residual adipose tissue on the prostatic surface. *Capsular incision *in this study was defined as an inked surgical margin involved by either benign prostatic glands or adenocarcinoma not associated with extraprostatic extension (EPE). For each case, the slides were microscopically examined and graded as 0 (no capsular incision), 1 (focal capsular incision) or 2 (capsular incision involving more than half of the inked surgical margin). We created a *capsular incision index *for each prostatectomy specimen, defined as the sum of scores from all the slides examined in one specimen divided by the highest possible sum of scores for the given specimen (i.e., 2 × number of slides). Residual adipose tissue on the prostate surface was evaluated by visual estimation of the percentage of inked prostate surface covered by adipose tissue in microscopic examination.

### Statistical analysis

Descriptive statistics included frequencies of categorical variables and means and standard deviations (SD) of quantitative variables. Associations between categorical variables were examined using Chi-square tests. Mean differences of quantitative variables between groups of cases were assessed using two-sample t-tests or analysis of variance. Correlation coefficients were calculated for some quantitative variables. All analyses were performed using SAS version 9. All tests were at a significance level α = 0.05.

## Results

### Demographics of prostate cancer patients with RALP and RRP procedures

The demographics of the 262 prostate cancer patients in this study who underwent prostatectomy by either RALP or RRP are listed in Table 1. Of these patients, 183 underwent RALP, representing 69.6% of the patients receiving prostatectomy for the treatment of prostate cancer during this period of time in our institution. The average ages of these two groups of patients were similar (60.8 years old for RALP and 60.5 years old for RRP). The pathologic stage and tumor volume did not differ significantly between the two groups (*P *= 0.305 and 0.207 respectively). Patients receiving RALP had lower average Gleason scores and greater average prostate weights (both statistically significant, *P *< 0.001).

**Table 1:** Demographics and comparison of positive surgical margins between RALP and RRP

	RALP	RRP	*P*
Case number	182	80	
Percentage	69.5%	30.5%	
Age	60.8 ± 6.8*	60.5 ± 6.8*	0.772**
Weight (gram)	47.8 ± 16.1*	40.1 ± 14.8*	< 0.001**
Gleason score	6.5 ± 0.8*	7.0 ± 0.8*	< 0.001**
Tumor volume (%)	16.0 ± 13.6*	18.8 ± 14.9*	0.207 **
Pathologic stage			0.305***
T2a	28 (15.4%)	6 (7.5%)	
T2b	8 (4.4%)	4 (5.0%)	
T2c	112 (61.5%)	51 (63.8%)	
T3	33 (18.1%)	19 (23.8%)	
T4	1 (0.6%)	0 (0.0%)	
Positive surgical margin (positive rate in parentheses)			
All patients	52 (28.6%)	46 (57.5%)	< 0.001**
T2a & T2b	8 (22.2%)	3 (30.0%)	0.610**
T2c	27 (24.1%)	30 (58.8%)	< 0.001**
T3 & T4	17 (50.0%)	13 (68.4%)	0.194**

* Mean ± SD; ** Student *t*-test for the analysis between RALP and RRP; *** Chi-square test (T4 not included in the test)

### Difference in surgical margin positivity rates between RALP and RRP

We first compared the surgical margins of the prostatectomy specimens for all patients involved in this study: while only 28.6% of the RALP group had positive surgical margins, 57.5% of the RRP group had positive surgical margins. The difference between these two groups was statistically significant (*P *< 0.001, Table 1). We also compared the surgical margins by pathologic stage between these two groups. For stage T2c patients, representing 61.5% of RALP patients and 63.8% of RRP patients, the positive surgical margin rate was 24.1% in the RALP group and 58.8% in the RRP group (statistically significant, *P *< 0.001). In patients with stages T2a or T2b, and these with T3 or T4, the difference in positive surgical margin rates between RALP and RRP groups was not statistically significant (*P *= 0.610 and 0.194 respectively, Table 1).

### Factors related to positive surgical margins in RALP

For prostatectomy performed by RALP, we compared the tumor stage, tumor volume, Gleason score and prostate weight between patients with positive surgical margins and those with negative surgical margins (Table 2). The result showed that the group with positive surgical margins was related with higher tumor stage, higher tumor volume, higher Gleason score and lower prostate weight (all statistically significant, *P *= 0.014, 0.021, 0.033 and 0.004 respectively). Positive surgical margin rates did not differ significantly among different surgeons performing RALP (*P *= 0.977, Table 2). Similar analyses performed in RRP specimens found that only higher tumor volume was related to positive surgical margins; other factors showed no statistically significant difference between margin-positive and margin-negative groups in the RRP cohort (results not shown).

**Table 2:** Factors associated with positive surgical margins in prostatectomy specimens from RALP

	Negative Margin	Positive Margin	*P*
Case number	130	52	
Tumor stage			0.014*
2a	23	5	
2b	5	3	
2c	83	27	
≥ 3a	17	17	
Tumor volume (%)	14.1 ± 11.5**	21.2 ± 17.2**	0.021***
Gleason score	6.5 ± 0.7**	6.8 ± 0.8**	0.033***
Weight (gram)	49.8 ± 16.6**	42.7 ± 13.7**	0.004***
Surgeons			0.977*
Surgeon one	37	14 (27.5%)****	
Surgeon two	69	28 (28.9%)****	
Others	24	10 (29.4%)****	

* Chi square test; ** Mean ± SD; *** Student *t*-test between negative surgical margin group and positive surgical margin group; **** Positive surgical margin rate for each surgeon in parentheses

### Differences in capsular incision index and prostate surface adipose tissue between RALP and RRP specimens

**Figure 1:**
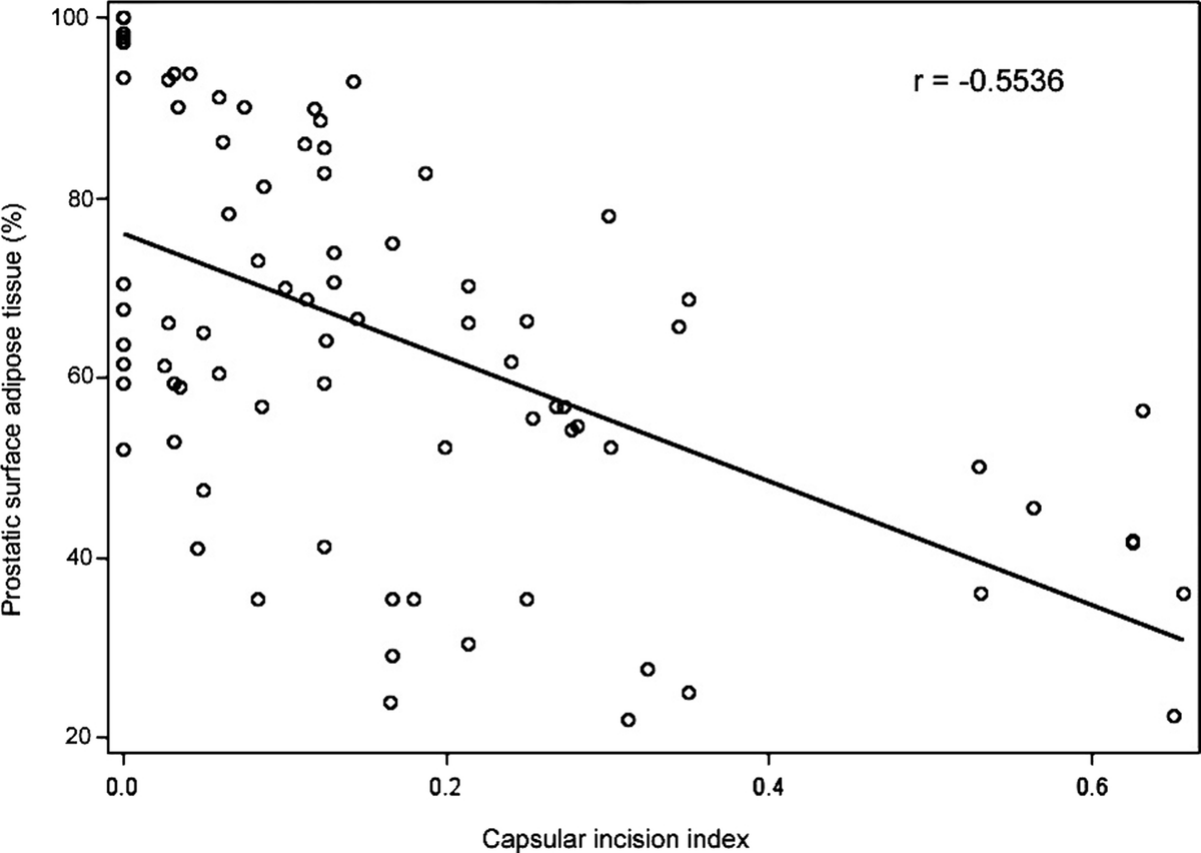
**Correlation between capsular incision index and the amount of residual prostatic surface adipose tissue**. The values of capsular incision and prostatic surface adipose tissue from 82 cases of prostatectomies (RALP and RRP) were analyzed for their correlation.

Capsular incision indicates damage to the prostate capsule caused by surgical procedures, which may contribute to a positive surgical margin if tumor is present at the site of capsular incision. As described in "Methods", we created a capsular incision index in this study to reflect the severity of capsular damage: a higher index suggests more extensive damage to the prostatic capsule during the surgical process. Analysis of 49 cases of RALP and 33 cases of RRP showed that the average capsular incision index was 0.127 for the RALP group and 0.233 for the RRP group (Table 3). The difference between these two groups was statistically significant (*P *= 0.009), suggesting that RRP caused more severe prostate capsular damage than RALP. We also compared the amount of residual surface adipose tissue in the RALP and RRP prostatectomy specimens, because the presence of surface adipose tissue suggests an undamaged prostate capsule at the observed area. Our analyses showed that the average percentage of prostatic surface covered with adipose tissue in RALP specimens was 73.2%, while that in RRP specimens was only 51.4% (Table 3), a statistically significant difference (*P *< 0.001). We also analyzed the correlation between capsular incision index and the percentage of surface adipose tissue and found these two factors to have a negative linear correlation, with an r = -0.5536 (*P *< 0.001, Figure 1). This finding was consistent with our prediction that both capsular incision index and amount of prostatic surface adipose tissue are indicators of the degree of prostate capsular damage caused by surgical procedures.

**Table 3:** Comparison of capsular incision index and residual prostatic surface adipose tissue between prostatectomy specimens from RALP and RRP

	Case number	Capsular incision Index	Surface adipose tissue (%)
RALP	49	0.127 ± 0.156	73.2 ± 21.0
RRP	33	0.233 ± 0.186	51.4 ± 15.6

		*P *= 0.009	*P *< 0.001

## Discussion

RALP evolved over the last decade with significant efforts to improve functional outcomes following prostatectomy and has greatly changed the standard treatment of prostate cancer. Currently, about 69-85% of radical prostatectomies performed in the Unites States are done robotically [4]. Our study showed that 69.6% of the prostatectomies performed at our institution from 2007 to 2010 were RALP, a proportion consistent with current national utilization rates of the procedure. In order to compare the RALP and RRP procedures, we analyzed their respective positive surgical margin rates and found that the RALP patients had a lower average positive surgical margin rate than the RRP patients. When only the patients with stage T2c disease were compared, the positive surgical margin rate was still lower in the RALP group, but in patients with other pathologic stages, the differences in positive surgical margin rates between RALP and RRP groups were not statistically significant (Table 1).

Previous reports comparing positive surgical margins between RALP and RRP had conflicting results: some showed no significant difference between RALP and RRP [8-11]; others found a higher incidence of positive surgical margins in RALP [12-14]; while many suggested that RALP resulted in a lower incidence of positive surgical margins than RRP. Ficarra et al. analyzed the cumulative data from six studies published before 2008, and found a statistically significant difference in favor of RALP [15]. More recently, Coelho et al. compared the data from published reports of prostatectomies performed in high-volume centers, including 11 studies of RALP (8,472 cases) and 19 studies of RRP (41,729 cases), and found that the weighted mean positive surgical margin rates were 13.6% for RALP and 24% for RRP [5]. Other studies have also shown lower positive surgical margin rates in RALP than in RRP [16,17]. It should be noted that all of the reports cited above were based on non-randomized studies, so that definitive conclusions can only be reached after randomized trials are done. When reviewing the previous studies, we noted that the positive surgical margin rate in our RALP group (28.6%) was comparable with the published data (9.3-33.3%) [5], but our RRP group showed a higher positive surgical margin rate (57.5%) than the reported range (8.8% to 42.8%) [5,16]. The reason for the high positive surgical margin rate in our RRP patients is not clear. When evaluating the difference of positive surgical margin rates between the RALP and RRP patients in our study, we should also consider that the unusually high positive surgical margin in our RRP group may partially contribute to such a significant difference. However, as an institutional experience, the introduction of RALP into our institute did result in significantly reduced positive surgical margin rate of prostatectomy.

In an effort to analyze the factors associated with positive surgical margins in RALP specimens, we compared the tumor stage, tumor volume, Gleason score and prostate weight between the patients with positive and negative surgical margins, and found that the patients with positive surgical margins after RALP had higher tumor stage, higher tumor volume, higher Gleason score and lower prostate weight than those with negative surgical margins (Table 2). Our findings are consistent with some previous studies that analyzed the risk factors associated with positive surgical margins after RALP. Liss et al. analyzed 216 consecutive cases of RALP and found that the pathological stage and final pathological Gleason score were the strongest positive surgical margin predictors, while clinical stage and biopsy Gleason score were not predictors of a positive surgical margin [18]. Similarly, the study by Ficarra et al. that included 322 consecutive cases of RALP found that pathological stage and Gleason score in the prostatectomy specimens were independent predictors of multiple positive surgical margins [19]. As for the association between prostate weight and positive surgical margins in RALP, Marchetti et al. analyzed 690 low-risk prostate cancer patients receiving RALP and found that smaller prostate weight was independently associated with a higher probability of positive surgical margins [20]. Several other studies also noted an association of smaller prostate weight with higher positive surgical margin rates in RALP [21-24]. Our study results support these findings.

In this study, we also evaluated the damage to the prostate capsule caused by surgical procedures. The term "capsular incision" has long been used to describe the situation in which "the surgeon inadvertently develops the plane of resection within the prostate rather than exterior to the prostate" [25]. More recently it has also been referred to in many literatures as "intraprostatic incision", because prostate is considered not to have a true capsule [26]. In organ-confined prostate cancer, capsular incision in the area of tumor may cause a positive surgical margin. On the other hand, capsular incision which involves only benign prostatic glands has been found to have no significant association with age, preoperative PSA, prostate weight, pathological stage, tumor volume, Gleason score, PSA recurrence and other prognostic factors [27], so that capsular incision can theoretically be an independent indicator for evaluating the capsular damage caused by different surgical procedures. For this study we created a "capsular incision index" to reflect the levels of prostate capsular damage. Because this index is the result of examination of all slides in a specimen and is not affected by the size of the prostate, it may more accurately reflect the severity of capsular damage than the measurement of the size of capsular incision as done in many previous studies. Our results show that the capsular incision index was lower in RALP than RRP, suggesting that a more intact prostate capsule was preserved in RALP. The presence of benign glands at surgical margins of RALP specimens has been previously studied in only a few reports with a small number of cases. In a study by Kohl et al. that included 25 cases of RALP and 13 cases of RRP, benign glands were present at the surgical margins of 54% RALP and 15% RRP, a statistically significant difference [28]. The discrepancy between Kohl's study and ours is difficult to analyze because both are based on institutional experience with relatively small numbers of cases, although the case numbers in our study are more than twice those of Kohl's.

Our study also found that prostatectomy specimens from RALP showed more residual adipose tissue on the prostate surface than those from RRP. This is another evidence to suggest a better-preserved prostate capsule after RALP, since prostate surface adipose tissue can only be seen in the area where the capsule is not damaged. The amount of adipose tissue present on the prostate surface after prostatectomy is not related to tumor stage, Gleason score, tumor volume or many other factors [29], so that it can also function as an independent indicator for the evaluation of prostate capsular damage caused by surgical procedures. Because the presence of surface adipose tissue is important for the diagnosis of extraprostatic extension (EPE) of prostate cancer [29,30], our findings also suggest that RALP may provide better prostatectomy specimens for pathologic staging of prostate cancer than RRP.

## Conclusions

In this study we analyzed 182 RALP and 80 RRP cases performed at our institution. We found that RALP performed in our institute caused statistically significant lower positive surgical margin rates than RRP, although we also noticed that the positive surgical margin rate of RRP in our study was higher than many previous reports. Our results also showed that positive surgical margin incidence after RALP was related to higher tumor stage, higher Gleason score, higher tumor volume and lower prostate weight. Analysis of capsular incision and the amount of residual adipose tissue on prostate surface in prostatectomy specimens suggested that RALP causes less capsular damage than RRP.

## Abbreviations

RALP: Robotic-assisted laparoscopic prostatectomy; RRP: Radical retropubic prostatectomy; PSA: Prostate-specific antigen.

## Competing interests

The authors declare that they have no competing interests.

## Authors' contributions

HH, LM, JT and HR contributed to study design, data collection and analysis. HH and LM contributed to additional microscopic examination. QW was responsible for the statistical analysis. All authors approved the current version of the manuscript.
